# Activity-Dependent Arc Expression and Homeostatic Synaptic Plasticity Are Altered in Neurons from a Mouse Model of Angelman Syndrome

**DOI:** 10.3389/fnmol.2017.00234

**Published:** 2017-07-28

**Authors:** Elissa D. Pastuzyn, Jason D. Shepherd

**Affiliations:** Department of Neurobiology and Anatomy, University of Utah Salt Lake City, UT, United States

**Keywords:** Arc, homeostatic scaling, Angelman syndrome, synaptic plasticity, hippocampus

## Abstract

Angelman syndrome (AS) is a neurodevelopmental disorder that results from deletions or mutations in chromosome 15, which usually includes the *UBE3A* gene. Ube3A protein is an E3 ubiquitin ligase that ubiquitinates proteins and targets them for degradation. The immediate-early gene Arc, a master regulator of synaptic plasticity, was identified as a putative substrate of Ube3A, but there have been conflicting reports on whether Arc is a bona fide E3 ligase substrate. Using multiple approaches, we found no evidence for a physical interaction between Arc and Ube3A *in vivo*. Nonetheless, activity-induced subcellular distribution of Arc is altered in brains from *Ube3a*^*m*−/*p*+^ mice, with abnormal concentration of Arc at synapses. Furthermore, although activation of Arc transcription is normal, the stability of Arc protein is enhanced in dendrites of hippocampal neurons cultured from *Ube3a*^*m*−/*p*+^ mice. Finally, homeostatic synaptic scaling of surface AMPA receptors does not occur in *Ube3a*^*m*−/*p*+^ hippocampal neurons, reminiscent of neurons that lack Arc protein. Although Ube3A does not seem to bind Arc in a canonical E3 ligase-substrate interaction, Arc-dependent synaptic plasticity is still altered in *Ube3a*^*m*−/*p*+^ mice, which may underlie the cognitive deficits observed in AS.

## Introduction

Angelman syndrome (AS) is a neurodevelopmental disorder affecting about one in 15,000 children, and presents with intellectual disability, ataxia, inability to acquire language and seizures (Clayton-Smith and Laan, 2003; Dagli et al., 2011). AS is caused by deletions or copy number variants in the region containing the maternally-inherited allele of the gene *UBE3A* on the 15q11.2-13 chromosome (Williams et al., 2006). This gene encodes the E3 ubiquitin ligase Ube3A (Kishino et al., 1997; Matsuura et al., 1997), which is imprinted in most parts of the brain (Albrecht et al., 1997; Rougeulle et al., 1997). As an E3 ligase, Ube3A catalyzes the addition of ubiquitin to proteins and thus tags them for degradation through the proteasome (Yi and Ehlers, 2005, 2007). A mouse model of AS, *Ube3a*^*m*−/*p*+^, has a deletion of the maternal allele of the *UBE3A* gene, while the paternal allele is still present and is epigenetically silenced in most brain regions (Jiang et al., 1998). AS mice recapitulate many of the same features as humans with AS, including gait and balance problems, deficits in context-dependent learning and seizures (Jiang et al., 1998; Miura et al., 2002; Yashiro et al., 2009).

Although a great deal is known about the genetics of AS, there is still little known about the normal role of Ube3A in brain development or function. Identifying potential substrates of Ube3A may help elucidate its role in the brain. Several non-neuronal (p53, annexin A1 and Ring1b; Huibregtse et al., 1991; Shimoji et al., 2009; Zaaroor-Regev et al., 2010) and neuronal (the potassium channel SK2 and the promyelocytic leukemia (PML) tumor suppressor; Louria-Hayon et al., 2009; Sun et al., 2015) substrates have been discovered, but finding neuronal-specific substrates that could explain Ube3A’s role in cognition has proven to be a challenge.

One study showed that Ube3A can bind to the neuronal protein Arc (Greer et al., 2010). Arc (activity-regulated, cytoskeleton-associated gene; Link et al., 1995; Lyford et al., 1995) is a highly dynamic immediate-early gene that is transcribed rapidly in response to activity, trafficked to dendrites and translated locally at synapses (Steward et al., 1998; Guzowski et al., 1999). There, Arc controls synaptic strength and homeostatic scaling via endocytosis of AMPA-type glutamate receptors (Chowdhury et al., 2006; Shepherd et al., 2006). Acute knockdown of Arc impairs memory consolidation and long-term potentiation (LTP; Guzowski et al., 2000; Messaoudi et al., 2007; Pastuzyn et al., 2012; Pastuzyn and Keefe, 2014). Arc knockout (KO) mice have normal short-term memory but severely impaired long-term memory (Plath et al., 2006) and long-term depression (LTD; Park et al., 2008; Waung et al., 2008). Furthermore, Arc KO mice have deficiencies in experience-dependent plasticity (Gao et al., 2010; McCurry et al., 2010) that are similar to those observed in *Ube3a*^*m*−/*p*+^ mice (Yashiro et al., 2009). The timing and expression of Arc protein is extremely sensitive to perturbations. Dysregulation of Arc expression has been implicated in many neurodevelopmental disorders. Tuberous sclerosis model mice have reduced Arc expression and fragile X syndrome model mice increased Arc, yet both models exhibit similar cognitive dysfunction. When these two models were crossed, both Arc expression and synaptic plasticity were normalized (Auerbach et al., 2011). These experiments suggest that either too much or too little Arc expression is detrimental for synaptic plasticity and cognition. Thus, if Ube3A directly regulates the degradation of Arc, Arc protein expression should be misregulated in AS. However, in contradiction with Greer et al. (2010), recent studies have shown that Arc and Ube3A do not physically interact (Kühnle et al., 2013; Mabb et al., 2014), although these studies and others (Cao et al., 2013) demonstrated that Arc expression is still dysregulated in *Ube3a*^*m*−/*p*+^ mice. Moreover, reducing Arc levels in *Ube3a*^*m*−/*p*+^ mice by crossing them to a heterozygous Arc KO mouse decreased seizure susceptibility, a hallmark phenotype of patients with AS (Mandel-Brehm et al., 2015). Arc may therefore play an important role in AS pathology and cognition, but the conflicting reports in the literature arising from variations in how experiments were performed have made it difficult to draw definitive conclusions about whether Arc is a proper Ube3A substrate. It is also unclear precisely how Arc protein misregulation could lead to plasticity and cognitive deficits in AS. In order to clarify the role of Arc in AS, we carried out a comprehensive set of experiments to determine whether Arc interacts with Ube3A *in vivo* and whether Arc protein expression and Arc-dependent synaptic plasticity are dysregulated in a mouse model of AS.

## Materials and Methods

### Animals

AS model mice (hybrid C57BL/6 and 129/SvEv background) were a generous gift from Yong-Hui Jiang (Jiang et al., 1998). Male *Ube3a*^*m*−/*p*+^ mice were bred to female WT C57BL/6J mice to obtain litters containing *Ube3a*^*m*+/*p*−^ and WT mice. Female mice lacking the paternal allele of *UBE3A* (*Ube3a*^*m*+/*p*−^) were then bred to male WT C57BL/6J mice (The Jackson Laboratory, Bar Harbor, ME, USA) to create litters containing *Ube3a*^*m*−/*p*+^ and WT (*Ube3a*^*m*+/*p*+^) mice on the C57 background. Both male and female *Ube3a*^*m*−/*p*+^ and WT mice were used. Arc KO mice were described previously (Wang et al., 2006). These studies were approved by and carried out in accordance with the recommendations of the Institutional Animal Care and Use Committee of the University of Utah.

### Antibodies

For immunocytochemistry, the following antibodies were used: rabbit anti-Arc (custom-made, ProteinTech, Rosemont, IL, USA); DAPI (Molecular Probes, Thermo Fisher Scientific, Waltham, MA, USA); mouse anti-GluA1-NT (custom-made, generous gift from Dr. Richard Huganir; Widagdo et al., 2015); chicken anti-MAP2 (ab5392, Abcam, Cambridge, MA, USA); Alexa Fluor 488-, 555- and 647-conjugated secondary antibodies raised in donkey (Thermo Fisher Scientific; Jackson ImmunoResearch, West Grove, PA, USA). For Western blots, the following antibodies were used: rabbit anti-Arc (custom-made, ProteinTech); rabbit anti-E6AP (Ube3A; A300-352A, Bethyl Laboratories, Montgomery, TX, USA); goat anti-E6-AP (sc-8926, Santa Cruz Biotechnology, Santa Cruz, CA, USA); mouse anti-PSD-95 (75-028, clone K28/43, UC Davis/NIH NeuroMab Facility, Davis, CA, USA); goat anti-rabbit-HRP (111-035-003, Jackson ImmunoResearch); donkey anti-goat-HRP (705-035-003, Jackson ImmunoResearch); goat anti-mouse-HRP (115-035-003, Jackson ImmunoResearch). For immunoprecipitations, normal rabbit or mouse IgG (sc-2027 or sc-2025, Santa Cruz) was used as a control.

### Enriched Environment

To induce Arc to a similar extent across mice and across experiments (Tagawa et al., 2005; Wang et al., 2006; Gao et al., 2010), mice used for immunoprecipitation and subcellular fractionation experiments were first housed in a sound- and light-proof animal chamber for 24 h, then taken out and exposed to light and novel objects placed in their cages for 2 h (“enriched environment” condition). Mice in the “basal” condition were euthanized in the animal facility in the morning of experiment days.

### Immunoprecipitation

Mice at the appropriate age were exposed to an enriched environment for 2 h before being sacrificed. Depending on the experiment, hippocampi or cortices were dissected out and homogenized in either immunoprecipitation lysis buffer (150 mM NaCl, 50 mM Tris-HCl, 0.5% Triton X-100, 0.05% sodium deoxycholate, pH 7.4) or synaptoneurosome buffer (in mM: 118 NaCl, 4.7 KCl, 1.2 MgSO_4_, 2.5 CaCl_2_, 1.53 KH_2_PO_4_, 212.7 D-glucose, 1 DTT, protease inhibitor, pH 7.4; Waung et al., 2008). An input sample was taken (10% of initial volume), then the appropriate antibody was added to the tissue lysate at a concentration of 1 μg/mL and rotated at 4°C for 2 h. Protein A beads (Thermo Fisher Scientific) were added to the lysate at 10% of the total lysate volume and rotated at 4°C for 1 h. The samples were then spun briefly, the unbound fraction removed, and beads washed three times in immunoprecipitation buffer. Protein was eluted from the beads with Laemmli buffer for 5 min at room temperature (RT), then denatured at 70°C for 10 min. Immunoprecipitation experiments from hippocampal and cortical lysate were performed in multiple independent replicates from multiple animals.

### Subcellular Fractionation

Cortical (excluding midbrain and hippocampus) or hippocampal tissue was homogenized in synaptoneurosome buffer with a glass dounce homogenizer (Kimble Chase, Rockwood, TN, USA), then frozen overnight at −20°C to assist in obtaining clean nuclear fractions (von Hungen et al., 1968). The sample was then thawed on ice, filtered consecutively through two 100-μm filters (Corning, Corning, NY, USA), and returned to the initial lysate volume (1 mL) with synaptoneurosome buffer. A sample of the lysate was taken (“input”; 10% of initial volume), then the remaining lysate was split into two aliquots to aid in recovery of sufficient synaptoneurosome sample to perform immunoprecipitation. One aliquot was centrifuged at 1000× *g* for 10 min at 4°C to isolate the nuclear pellet, which was resuspended in synaptoneurosome buffer for Western blotting (200 μL) or in immunoprecipitation buffer for immunoprecipitation (600 μL). The other aliquot was filtered through a 5-μm filter (UFC30SV00, Millipore, Billerica, MA, USA) at 12,000× *g* to isolate the synaptoneurosome pellet (Waung et al., 2008), which was resuspended like the nuclear pellet depending on experiment. Subcellular fractionation experiments were performed in multiple independent replicates from multiple animals.

### Western Blotting

Protein samples were denatured in Laemmli buffer at 70°C for 10 min, then separated by SDS-PAGE. Separated samples were transferred to a nitrocellulose membrane (GE Healthcare, Pittsburgh, PA, USA). Total protein in each lane was detected using either Ponceau stain for immunoprecipitations, or the Pierce reversible protein stain kit (Thermo Fisher Scientific) for synaptoneurosome Western blots, then imaged using an Azure c300 gel dock (Azure Biosystems, Dublin, CA, USA). Membranes were blocked in 5% milk + 1× tris-buffered saline (TBS; 10×: 152.3 mM Tris-HCl, 46.2 mM Tris base, 1.5 M NaCl, pH 7.6) for 30 min at RT, then incubated in primary antibody in 1× TBS for either 1 h at RT or overnight at 4°C. Membranes were washed 3 × 10 min in 1× TBS, then incubated in an HRP-conjugated secondary antibody in block for 1 h at RT. After 3 × 10 min in 1× TBS, a chemiluminescent kit (Bio-Rad, Hercules, CA, USA) was used to detect the protein bands, and the membranes were imaged on a c300 gel dock.

### Western Blot Analysis

Immunoblotted membranes were analyzed using the gel analysis plugin in ImageJ (National Institutes of Health, Bethesda, MD, USA). Values for bands for Ube3A and Arc were normalized to the total protein, determined by the Pierce reversible protein stain kit, in each lane. The efficacy of the synaptoneurosome preparation was determined by the reversible protein stain, and if no total protein was observed, then that sample was removed from subsequent analysis (in Figure 2, one out of four samples).

**Figure 1:**
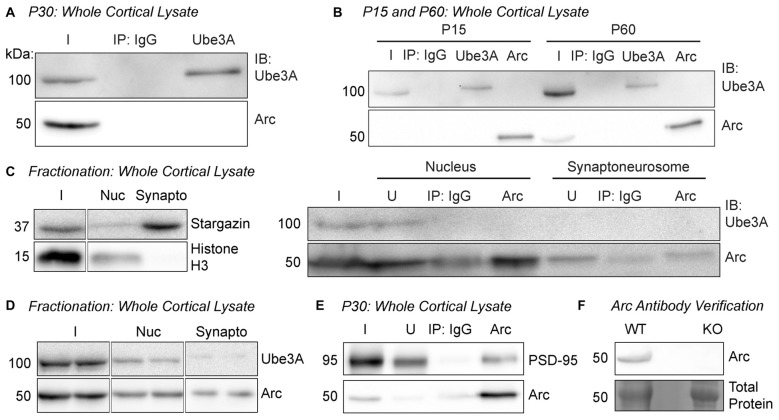
Arc and Ube3A do not interact in the cortex *in vivo*.** (A)** Ube3A was immunoprecipitated from cortical lysate of P30 WT mice. Immunoblots for Arc and Ube3A show that Arc does not coimmunoprecipitate with Ube3A. **(B)** Arc or Ube3A were immunoprecipitated from cortical lysates of P15 or P60 WT mice. No coimmunoprecipitations were observed at either age. **(C)** Subcellular fractionation was performed on cortical lysate from P30 WT mice to isolate the nucleus and the synaptoneurosome compartments. The blot shown is a representative of all fractionation experiments. Left, efficacy of subcellular fractionation. The synaptic protein Stargazin was enriched in the synaptoneurosome fraction, and the nuclear protein histone H3 was enriched in the nucleus. Right, Arc was immunoprecipitated from the nuclear and synaptoneurosome fractions. Ube3A did not coimmunoprecipitate in either subcellular compartment. **(D)** Subcellular fractions from cortical lysate from P30 WT mice were immunoblotted for Ube3A and Arc. Arc was present in both fractions, while Ube3A expression at the synapse was very low. **(E)** Arc was immunoprecipitated from cortical lysate of P30 WT mice. PSD-95 coimmunoprecipitated with Arc. **(F)** Our custom Arc antibody detects protein at the predicted molecular weight for Arc in lysate from WT cultured hippocampal neurons and no detectable band is observed in Arc knockout (KO) lysate. I, input; IB, immunoblot; nuc, nucleus; synapto, synaptoneurosome; U, unbound fraction.

**Figure 2:**
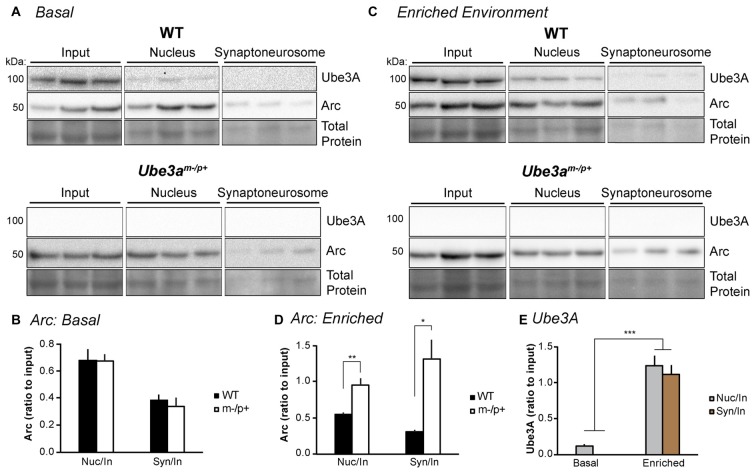
Experience alters Arc subcellular localization in the hippocampus of *Ube3a*^*m*−/*p*+^ mice.** (A)** WT and *Ube3a*^*m*−/*p*+^ mice (*n* = 3/group) were euthanized under “basal” conditions in the animal facility. Subcellular fractionation was performed on hippocampal lysates to isolate the nucleus and synaptoneurosome. “Input” is lysate sample prior to fractionation. Samples were separated by SDS-PAGE gel electrophoresis and immunoblotted for Arc and Ube3A. **(B)** Arc bands were analyzed and normalized to total protein in each lane. The ratios of nucleus:input (“nuc/in”) and synaptoneurosome:input (“syn/in”) Arc were identical between WT and *Ube3a*^*m*−/*p*+^ mice. **(C)** Mice were placed in the dark for 24 h to normalize activity, then exposed to an enriched environment in the light for 2 h before sacrifice, subcellular fractionation, and Western blot analysis. **(D)** The nucleus:input and synaptoneurosome:input ratios of Arc expression were significantly higher in *Ube3a*^*m*−/*p*+^ mice than WT. *P < 0.05, **P < 0.01. **(E)** Ube3A expression was analyzed in blots from WT mice in **(A,C)**. Ube3A levels were significantly higher in nuclear and synaptoneurosome fractions after exposure to an enriched environment. ***P < 0.0001.

### Neuron Culture

The neuron culture protocol was based on Shepherd et al. (2006). Hippocampi were dissected from E18 Arc KO or WT mouse embryos to test the antibody, or E18 WT and *Ube3a*^*m*−/*p*+^ mouse embryos for the experiments in Figures 4–7. Because the *Ube3a*^*m*−/*p*+^ litters contained both genotypes, hippocampi from individual mice were cultured separately, and embryos genotyped after the culture. Hippocampi were dissociated in DNase (0.01%; Sigma-Aldrich, St. Louis, MO, USA) and papain (0.067%; Worthington Biochemicals, Lakewood, NJ, USA), then triturated with a fire-polished glass pipette to obtain a single-cell suspension. Cells were pelleted at 1000× *g* for 4 min, the supernatant removed, and cells resuspended and counted with a TC-20 cell counter (Bio-Rad). Neurons were plated on glass coverslips (Carolina Biological Supply, Burlington, NC, USA) coated with poly-L-lysine (0.2 mg/mL; Sigma-Aldrich) in 12-well plates (Greiner Bio-One, Monroe, NC, USA) at 100,000 cells/mL. Neurons were initially plated in Neurobasal media containing 5% horse serum, 2% GlutaMAX, 2% B-27, and 1% penicillin/streptomycin (Thermo Fisher Scientific) in a 37°C incubator with 5% CO_2_. On DIV4, neurons were fed via half media exchange with astrocyte-conditioned Neurobasal media containing 1% horse serum, GlutaMAX and penicillin/streptomycin, 2% B-27, and 5 μM cytosine β-D-arabinofuranoside (AraC; Sigma-Aldrich). Neurons were fed with astrocyte-conditioned media every 3 days thereafter.

**Figure 3:**
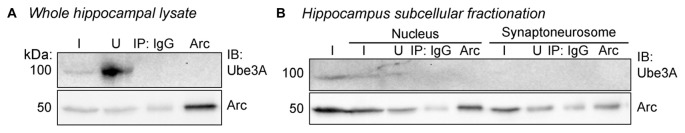
Arc and Ube3A do not interact in the hippocampus *in vivo*.** (A)** Arc was immunoprecipitated from whole hippocampal lysate from P30 WT mice and immunoblotted for Arc and Ube3A. Arc and Ube3A do not coimmunoprecipitate. **(B)** Hippocampal lysates from P30 WT mice underwent subcellular fractionation and Arc was immunoprecipitated from the nuclear and synaptoneurosome fractions. Arc and Ube3A do not coimmunoprecipitate. I, input; IB, immunoblot; nuc, nucleus; synapto, synaptoneurosome; U, unbound fraction.

**Figure 4:**
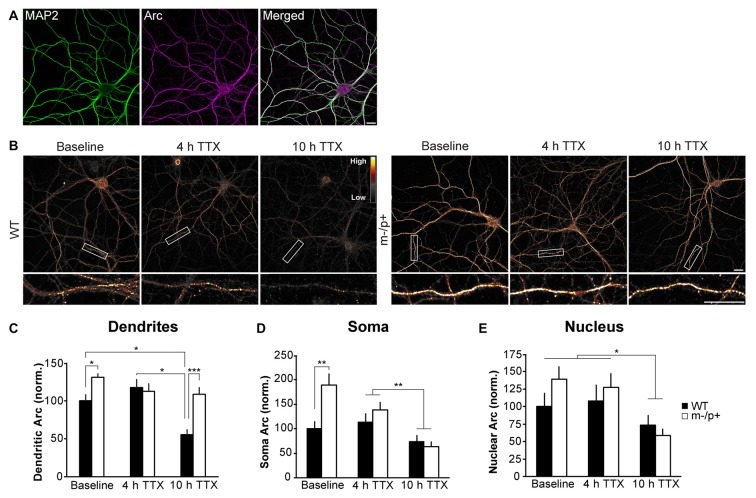
Arc protein is more stable in dendrites of *Ube3a*^*m*−/*p*+^ cultured neurons. Hippocampal neurons were cultured from E18 WT and *Ube3a*^*m*−/*p*+^ mice. At DIV19–21, neurons were treated with tetrodotoxin (TTX) for 4 or 10 h. Neurons were immunostained for Arc and expression analyzed in dendrites, cytoplasm and nucleus (*n* = 15 neurons/group, two dendrites/neuron). **(A)** A representative image of MAP2 and Arc immunostaining in a cultured hippocampal neuron. MAP2 was used to choose healthy neurons for imaging in all culture experiments. **(B)** Images of representative neurons from WT and *Ube3a*^*m*−/*p*+^ cultures at baseline or after 4 or 10 h TTX. To highlight intensity levels, images are shown using the Smart look up table (LUT) generated using ImageJ software. **(C)** In dendrites, Arc expression was significantly higher in *Ube3a*^*m*−/*p*+^ neurons than WT at baseline. Arc expression declined at 10 h TTX compared to 4 h and baseline in WT neurons, but not in *Ube3a*^*m*−/*p*+^ neurons. Arc levels were higher in *Ube3a*^*m*−/*p*+^ neurons than WT at 10 h TTX. **(D)** In the cytoplasm, Arc was significantly higher in *Ube3a*^*m*−/*p*+^ neurons than WT at baseline, but after 10 h TTX, Arc expression had significantly decreased in both genotypes. **(E)** In the nucleus, Arc was lower at 10 h TTX than baseline or 4 h, but there were no genotype differences. Scale bar = 10 μm. *P < 0.05, **P < 0.01, ***P < 0.001.

**Figure 5:**
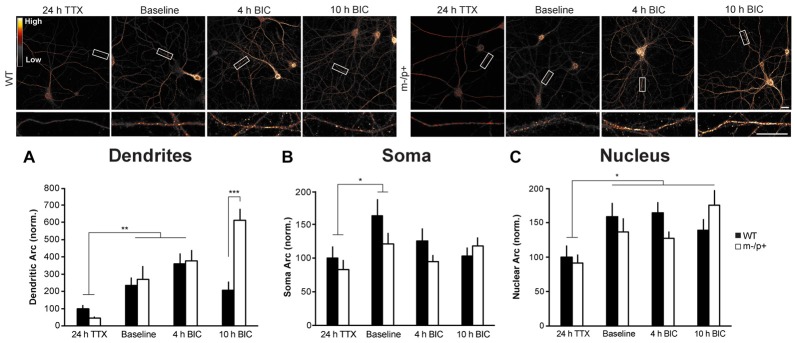
Arc induction is normal in dendrites of *Ube3a*^*m*−/*p*+^ neurons. Cultured hippocampal *Ube3a*^*m*−/*p*+^ and WT neurons at DIV19–21 were treated with TTX for 24 h to normalize activity, or left under basal (“Baseline”) conditions, then treated with bicuculline (BIC) for 4 or 10 h to induce activity and Arc expression. Neurons were immunostained for Arc and expression analyzed (*n* = 15 neurons/group, two dendrites/neuron). **(A)** In dendrites, after 24 h TTX, 4 h BIC significantly increased Arc in both genotypes. At 10 h BIC, *Ube3a*^*m*−/*p*+^ neurons expressed significantly more Arc than WT. **(B,C)** BIC significantly induced Arc expression in the nucleus, but not the cytoplasm, and there were no genotype differences in Arc induction in either compartment. Scale bar = 10 μm. *P < 0.05, **P < 0.01.

**Figure 6:**
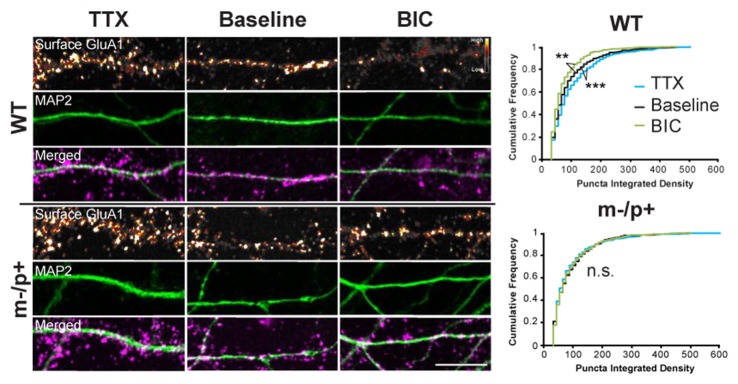
Homeostatic scaling of AMPA receptors is disrupted in *Ube3a*^*m*−/*p*+^ neurons. Cultured hippocampal *Ube3a*^*m*−/*p*+^ and WT neurons at DIV19–21 were treated with either TTX or BIC for 24 h to induce homeostatic scaling. Neurons were live-labeled for surface GluA1 (*n* = 15 neurons/group, two dendrites/neuron). BIC and TTX induced scaling of GluA1 in WT neurons, as demonstrated by a decrease or increase, respectively, in the cumulative frequency of GluA1 puncta integrated density. BIC and TTX-induced scaling was absent, however, in *Ube3a*^*m*−/*p*+^ neurons. Surface GluA1: smart LUT; MAP2: green; Merged: GluA1 magenta, MAP2 green. Scale bar = 10 μm. **P < 0.01, ***P < 0.001.

**Figure 7:**
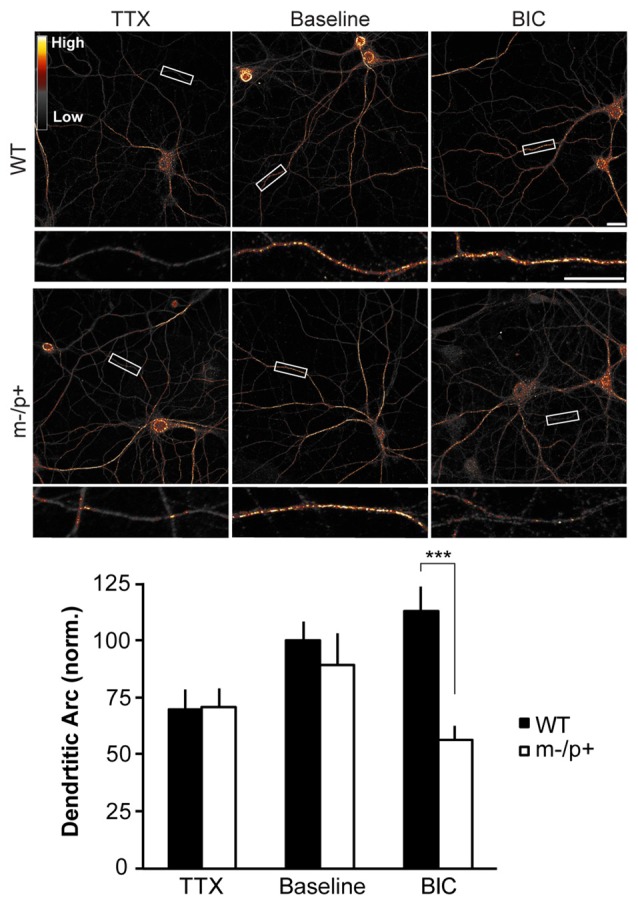
Homeostatic expression of Arc is disrupted in *Ube3a*^*m*−/*p*+^ neurons. Cultured hippocampal *Ube3a*^*m*−/*p*+^ and WT neurons at DIV19–21 were treated with either TTX or BIC for 24 h to induce homeostatic scaling. Neurons were immunostained for Arc and dendritic expression analyzed (*n* = 15 neurons/group, two dendrites/neuron). BIC and TTX induced changes in Arc expression in WT neurons, with an increase in Arc expression with BIC and a decrease with TTX. BIC-induced scaling was absent, however, in *Ube3a*^*m*−/*p*+^ neurons, and Arc levels were significantly lower than in WT neurons. Scale bar = 10 μm. ***P < 0.001.

### Drug Treatment

At DIV18–20, neuron cultures were treated with 1 μM tetrodotoxin (TTX; Abcam) or 10 μM bicuculline (BIC; Sigma-Aldrich; Shepherd et al., 2006) for durations as outlined in the results and figure legends.

### Immunocytochemistry

At DIV19–21, neurons used for Arc induction and degradation experiments were washed twice with 37°C 4% sucrose/1× phosphate-buffered-saline (PBS; 10×: 1.4 M NaCl, 26.8 mM KCl, 62 mM Na_2_HPO_4_, 35.3 mM KH_2_PO_4_, pH 7.4), then fixed for 15 min with 4% sucrose/4% formaldehyde (Thermo Fisher Scientific) in 1× PBS. Neurons were washed 3 × 5 min with 1× PBS, permeabilized for 10 min with 0.2% Triton X-100 (Amresco, Solon, OH, USA) in 1× PBS, and blocked for 30 min in 5% normal donkey serum (Jackson ImmunoResearch) in 1× PBS. Neurons were then incubated in primary antibody diluted in block for 1 h at RT, washed 3 × 5 min in 1× PBS, and incubated in secondary antibody diluted in block for 1 h at RT. Neurons on coverslips were mounted on glass slides in Fluoromount (Thermo Fisher Scientific) and dried overnight at RT. For live-labeling of surface GluA1 receptors (Shepherd et al., 2006), neurons were washed twice with 10°C 4% sucrose/1× PBS, then incubated in anti-GluA1-NT diluted in MEM containing 2% GlutaMAX, 2% B-27, 15 mM HEPES (Thermo Fisher Scientific), 1 mM sodium pyruvate (Thermo Fisher Scientific), and 33 mM glucose at 10°C for 20 min. Neurons were then fixed and incubated in Alexa Fluor 555 before permeabilization to label only surface GluA1. Following this, neurons were permeabilized and further immunostained as above.

### Neuron Imaging and Analysis

Fifteen neurons per condition were imaged at 60× on an Olympus FV1000 confocal microscope (Tokyo, Japan). Healthy neurons were chosen based on MAP2 immunostaining. Arc and GluA1 immunostaining were analyzed using ImageJ software. The most intense immunostaining in each condition was used to set an arbitrary pixel intensity threshold, which was applied to every image in the experiment. Thresholds were set to ensure that pixel values were in the linear range for all treatment groups with both WT and *Ube3a*^*m*−/*p*+^ neurons. For dendritic Arc, integrated density of a 30-μm segment on two secondary dendrites/neuron was quantified. For analyzing Arc in the soma, the integrated density of the whole soma and the nucleus alone were measured, and then nuclear integrated density was subtracted from soma integrated density to isolate cytoplasmic Arc integrated density. For surface GluA1, integrated density of each puncta in two 30-μm dendrite segments/neuron was measured and summed to obtain a total integrated density of the puncta on the dendritic segment. Data was normalized to the baseline condition in WT neurons in each experiment.

### Statistics

One- or two-way analysis of variances (ANOVAs) were performed for each experiment as described in the text using JMP Pro statistical software (v12; SAS, Cary, NC, USA). Student’s *t*-tests or Tukey HSD tests were used to make *post hoc* comparisons.

## Results

### Arc and *Ube3A* Do Not Interact in Young or Adult Mouse Brain

In order to determine whether Arc and Ube3A interact *in vivo* (Figure 1), we conducted coimmunoprecipitation experiments from brain tissue. Since basal Arc levels can be variable, to normalize and then induce Arc expression *in vivo*, we first dark-housed juvenile P30 WT mice (littermates of *Ube3a*^*m*−/*p*+^ mice) for 24 h, then exposed them to an enriched environment (see “Materials and Methods” Section) for 2 h before euthanasia (Tagawa et al., 2005; Wang et al., 2006; Gao et al., 2010). To ensure that we could obtain enough protein for immunoprecipitation, we dissected cortex from these mice and used an antibody against Ube3A (Bethyl Laboratories) for immunoprecipitation, followed by immunoblotting for Ube3A (Santa Cruz Biotechnology) and Arc (custom-made antibody, ProteinTech). Ube3A was successfully immunoprecipitated from cortical tissue, but Arc did not coimmunoprecipitate (Figure 1A), consistent with recent reports (Kühnle et al., 2013; Mabb et al., 2014). The Arc-Ube3A interaction may be developmentally regulated, as Ube3A expression changes during early postnatal development (Sato and Stryker, 2010; Judson et al., 2014). We therefore tested whether an Arc-Ube3A interaction was age-specific. We immunoprecipitated Ube3A or Arc from P15 or P60 WT mouse cortex (Figure 1B). Arc did not coimmunoprecipitate with Ube3A at either age. The distribution of Ube3A in neurons also changes during development, becoming more concentrated in the nucleus and less synaptic after age P7 (Judson et al., 2014; Burette et al., 2017). Thus, we determined whether an Arc-Ube3A interaction might be cell compartment-specific. We performed subcellular fractionation on P30 WT mouse cortex to isolate the nucleus and synaptoneurosome compartments and immunoprecipitated Arc from these fractions (Figure 1C). Ube3A did not coimmunoprecipitate with Arc in either fraction. We also noticed that Ube3A is only weakly expressed in the synaptoneurosome fraction at P30 (Figure 1D), suggesting that Ube3A expression in cortical synapses, even under conditions of an enriched environment, is low (Burette et al., 2017). This potentially explains the lack of a direct robust Arc-Ube3A interaction. PSD-95, a known binding partner of Arc (Cao et al., 2013; Nair et al., 2017) robustly coimmunoprecipitated with Arc in cortical lysate from P30 WT mice under the same conditions, suggesting that our immunoprecipitation conditions should be able to detect an Arc/Ube3A interaction (Figure 1E). Our custom-made Arc antibody detected a band at the appropriate molecular weight for Arc in lysate from WT cultured hippocampal neurons, and no band was detected in Arc KO lysate (Figure 1F). Together, these results show that Arc and Ube3A do not physically associate *in vivo*.

### Subcellular Localization of Arc Is Altered after Experience in *Ube3a*^*m*−/*p*+^ Mice

Despite the lack of interaction between Ube3A and Arc, dysregulation of Arc protein has been a consistent finding in *Ube3a*^*m*−/*p*+^ neurons (Greer et al., 2010; Cao et al., 2013; Kühnle et al., 2013; Mabb et al., 2014). Thus, we set out to determine, in detail, the Arc expression profile when Ube3A is absent in the brain. We first determined whether basal or induced subcellular Arc localization is abnormal *in vivo* in *Ube3a*^*m*−/*p*+^ mice. We performed subcellular fractionation on hippocampal lysates from *Ube3a*^*m*−/*p*+^ mice or WT littermates (*n* = 3/group). Mice were either sacrificed in the animal colony (“basal” condition), or dark-housed for 24 h to normalize activity, then exposed to light and novel objects (“enriched environment” condition) for 2 h before sacrifice. Hippocampi were dissected out, homogenized and subcellular fractionation performed to isolate the nucleus and synaptoneurosome. Lysates were immunoblotted for Arc and Ube3A (Figure 2). Under basal conditions (Figures 2A,B), the ratio of Arc in the nucleus to the input (“nuc/in”) and synaptoneurosome to the input (“syn/in”) was the same between WT and *Ube3a*^*m*−/*p*+^ mice (*Ube3a*^*m*−/*p*+^ nuc/in: 0.67 ± 0.05, WT nuc/in: 0.68 ± 0.08; one-way ANOVA, *F*_(1,4)_ = 0.005, *p* = 0.95; *Ube3a*^*m*−/*p*+^ syn/in: 0.34 ± 0.06, WT syn/in: 0.38 ± 0.04; one-way ANOVA, *F*_(1,4)_ = 0.43, *p* = 0.55). However, after exposure to an enriched environment (Figures 2C,D), Arc was concentrated more at the synapse and in the nucleus in *Ube3a*^*m*−/*p*+^ mice than WT (*Ube3a*^*m*−/*p*+^ nuc/in: 0.95 ± 0.09, WT nuc/in: 0.29 ± 0.03; one-way ANOVA, *F*_(1,4)_ = 51.43, *p* = 0.002; *Ube3a*^*m*−/*p*+^ syn/in: 1.31 ± 0.27, WT syn/in: 0.18 ± 0.03; one-way ANOVA, *F*_(1,4)_ = 18.11, *p* = 0.013). This suggests that activity-dependent Arc subcellular localization is abnormal in *Ube3a*^*m*−/*p*+^ mice. Interestingly, we found that expression of Ube3A also changes dramatically after activity (Figure 2E). Under basal conditions, Ube3A was present in the nucleus but was not detectable in the synaptoneurosome fraction. After exposure to an enriched environment, Ube3A levels increased significantly and could now be detected in synaptoneurosomes (main effect of condition (basal/light) by two-way ANOVA, *F*_(1,2)_ = 137.35, *p* < 0.0001). We therefore performed immunoprecipitation from both whole hippocampal lysate as well as fractionated hippocampal lysate to ensure that we were not diluting out a potential Arc-Ube3A interaction (Figure 3). Under these conditions, we were still unable to detect coimmunoprecipitation of Arc and Ube3A.

### Arc Protein Is More Stable in Dendrites of *Ube3a*^*m*−/*p*+^ Neurons

Despite a lack of a physical interaction with Ube3A, we set out to determine whether the stability of Arc protein is altered in *Ube3a*^*m*−/*p*+^ mice by utilizing an *in vitro* model of cultured hippocampal neurons from *Ube3a*^*m*−/*p*+^ and WT mice. Previous studies have shown that epigenetic silencing of the paternal allele is maintained in primary cultured neurons (Huang et al., 2012). We note that our cultures exhibit high levels of basal neuronal activity and Arc levels. These levels vary between independent cultures; therefore, we repeated experiments in at least three different cultures to ensure that cellular phenotypes were not an artifact of subtle differences between cultures. This was especially evident in basal Arc levels, which varied considerably in *Ube3a*^*m*−/*p*+^ and WT neurons; thus, phenotypes were more robust when activity levels were normalized with the use of pharmacological manipulations that affected global neuronal activity levels. Arc protein has a very short half-life of about 30 min (Mabb et al., 2014), suggesting that protein expression is highly dynamic. We treated DIV19–21 *Ube3a*^*m*−/*p*+^ and WT neurons with 1 μM TTX for 4 or 10 h to silence activity in the culture to prevent new synthesis of Arc, and thus examine the stability of existing Arc protein (Steward et al., 1998; Shepherd et al., 2006). Neurons were fixed, permeabilized, and immunostained for Arc (Figure 4). Neurons were chosen for analysis in a blinded manner based on MAP2 immunostaining (Figure 4A). Arc expression was analyzed in dendrites, cytoplasm and nucleus (*n* = 15 neurons/treatment group, two 30-μm segments of dendrites analyzed/neuron; Figure 4B). A two-way ANOVA revealed a main effect of genotype (*F*_(1,84)_ = 15.74, *p* = 0.0002), treatment (*F*_(2,84)_ = 8.35, *p* = 0.0005), and a significant genotype × treatment interaction (*F*_(1,2)_ = 5.67, *p* = 0.005; Figure 4C). At baseline (no TTX treatment), *Ube3a*^*m*−/*p*+^ neurons expressed more Arc than WT neurons (*Ube3a*^*m*−/*p*+^: 130.91 ± 4.79, WT: 100 ± 8.12; Tukey HSD, *p* = 0.034). Arc levels were not significantly different from baseline in either genotype at 4 h (*Ube3a*^*m*−/*p*+^: 112.36 ± 10.4, Tukey HSD, *p* = 0.7; WT: 117.18 ± 11.07; Tukey HSD, *p* = 0.4). By 10 h, Arc levels had declined significantly in WT neurons (55.23 ± 7.37; Tukey HSD, *p* = 0.049), but remained unchanged from baseline in *Ube3a*^*m*−/*p*+^ neurons (108.12 ± 9.63; Tukey HSD, *p* = 0.5). Furthermore, Arc levels in *Ube3a*^*m*−/*p*+^ neurons were higher than WT at 10 h (Tukey HSD, *p* = 0.001). When examining cytoplasmic levels of Arc in isolation from the nucleus (“soma”, Figure 4D; *n* = 13–15 neurons/group), there was a significant main effect of genotype (*F*_(1,82)_ = 7.08, *p* = 0.009), treatment (*F*_(2,82)_ = 11.81, *p* < 0.0001), and a genotype × treatment interaction (*F*_(1,2)_ = 4.99, *p* = 0.009). Arc levels were lower at 10 h than 4 h regardless of genotype (Tukey HSD, *p* = 0.002), although Arc expression was significantly higher in *Ube3a*^*m*−/*p*+^ neurons than WT at baseline (*Ube3a*^*m*−/*p*+^: 189.51 ± 22.28, WT: 100 ± 15.59; Tukey HSD, *p* = 0.002). Nuclear Arc (Figure 4E; *n* = 15 neurons/treatment group) levels did not exhibit any significant genotype differences. A two-way ANOVA showed that there was a significant main effect of treatment (*F*_(2,84)_ = 6.64, *p* = 0.002), but no effect of genotype (*F*_(1,84)_ = 0.32, *p* = 0.57) and no interaction (*F*_(1,2)_ = 0.63, *p* = 0.54). Arc expression at 10 h was significantly lower than at 4 h (Tukey HSD, *p* = 0.016) and baseline (*p* = 0.003). These data suggest that the stability of Arc protein is selectively altered in the dendritic compartment.

### Arc Induction Is Normal in *Ube3a*^*m*−/*p*+^ Neurons

The higher levels of Arc in *Ube3a*^*m*−/*p*+^ dendrites could either be due to altered Arc protein stability, as the results above suggest, or due to enhanced activity-dependent induction (transcription and/or translation) of Arc expression. To determine whether enhanced activity-dependent Arc induction occurs in *Ube3a*^*m*−/*p*+^ neurons, we treated DIV19–21 cultured hippocampal neurons with TTX for 24 h to normalize basal neuronal activity and Arc levels. We then replaced the culture media with fresh conditioned media containing 10 μM BIC for either 4 or 10 h to induce Arc. Neurons were then fixed, permeabilized, and immunostained for Arc, and two 30-μm segments of dendrite/neuron were analyzed (*n* = 15 neurons/treatment group; Figure 5A). A two-way ANOVA revealed a significant main effect of genotype (*F*_(1,84)_ = 13.09, *p* = 0.0005), treatment (*F*_(2,84)_ = 39.09, *p* < 0.0001), and an interaction (*F*_(1,2)_ = 17.65, *p* < 0.0001). *Post hoc* Tukey HSD tests showed that while Arc levels were not significantly different between WT and *Ube3a*^*m*−/*p*+^ neurons at 24 h TTX (*Ube3a*^*m*−/*p*+^: 46.9 ± 7.94, WT: 100 ± 21.05; *p* = 0.9) or at 4 h BIC (*Ube3a*^*m*−/*p*+^: 377 ± 63.48, WT: 360.57 ± 28.04; *p* = 0.9), at 10 h BIC, *Ube3a*^*m*−/*p*+^ neurons expressed significantly more Arc than WT (*Ube3a*^*m*−/*p*+^: 612.01 ± 66.6, WT: 207.56 ± 24.03; *p* < 0.0001). At 10 h BIC, WT neurons expressed less Arc than at 4 h (*p* = 0.04). Interestingly, and mirroring the results from the TTX experiment above, the difference in Arc expression between genotypes was only observed in dendrites. In the cytoplasm (Figure 5B), there were no significant effects or an interaction (two-way ANOVA, *p* > 0.05). In the nucleus (Figure 5C), there was a significant main effect of treatment (*F*_(2,84)_ = 8.79, *p* = 0.0003). BIC treatment increased Arc expression over the 24 h TTX baseline (Tukey HSD, 10 h BIC: *p* = 0.0005, 4 h BIC: *p* = 0.005). In this experiment, we did not observe a significant difference in basal dendritic Arc levels in *Ube3a*^*m*−/*p*+^ neurons (Figure 5A), unlike the results from the previous experiment (Figure 4C). When all experiments are combined, we did not observe a significant difference in basal Arc expression between genotypes due variability across cultures. As noted above, this is most likely explained by variability in activity levels across cultures. These results suggest that Arc is initially induced normally in *Ube3a*^*m*−/*p*+^ neurons, but that increased stability of Arc protein may result in accumulation of Arc in dendrites after induction.

### Homeostatic Scaling of GluA1 and Arc Is Disrupted in *Ube3a*^*m*−/*p*+^ Neurons

*Ube3a*^*m*−/*p*+^ mice have deficits in synaptic and experience-dependent plasticity that are thought to mimic deficits found in human patients with AS (Jiang et al., 1998; Yashiro et al., 2009). The loss of homeostatic regulation of neuronal output has been hypothesized to be a cardinal phenotype of many neurodevelopmental disorders (Zoghbi and Bear, 2012). Arc regulates homeostatic synaptic scaling of the AMPA-type glutamate receptors (AMPARs) in neurons by regulating the trafficking of GluA1 (Shepherd et al., 2006), a process of homeostatic plasticity that results in equal changes of synaptic strength at all synapses to compensate for prolonged levels of high or low neuronal activity (Turrigiano, 2012). We subjected DIV19–21 cultured hippocampal neurons from *Ube3a*^*m*−/*p*+^ or WT mice to chronic changes in neuronal activity to determine whether there was an AMPAR scaling defect in *Ube3a*^*m*−/*p*+^ neurons (Figure 6). Neurons were treated with 1 μM TTX or 10 μM BIC, or were left untreated for the “baseline” condition, for 24 h. Neurons were then live-labeled with an antibody against the N-terminus of GluA1 to label surface AMPARs (Shepherd et al., 2006). Neurons were then fixed, permeabilized and immunostained for Arc. GluA1 and Arc were analyzed in two 30-μm dendritic segments per neuron (*n* = 15 neurons/treatment group). The cumulative frequency of the integrated density of the GluA1 puncta was graphed and statistically analyzed using the Kolmogorov-Smirnov test. At baseline, there was no difference between the cumulative frequency distribution in WT and *Ube3a*^*m*−/*p*+^ neurons (*p* = 0.5). In WT, as expected, 24 h TTX increased (upscaling) and 24 h BIC decreased (downscaling) the cumulative frequency of surface GluA1 puncta integrated density (Baseline vs. TTX: *p* = 0.008; Baseline vs. BIC: *p* < 0.0001). Strikingly, TTX and BIC were unable to induce homeostatic scaling in *Ube3a*^*m*−/*p*+^ neurons, with no change in the distribution of surface GluA1 puncta integrated density (Baseline vs. TTX: *p* = 0.49; Baseline vs. BIC: *p* = 0.88). One explanation for this result is that Arc expression is uncoupled from neuronal activity in *Ube3a*^*m*−/*p*+^ neurons. To test this hypothesis, we measured Arc levels in the same neurons that had undergone homeostatic scaling to determine whether Arc expression correlated with the lack of BIC-induced GluA1 scaling observed in *Ube3a*^*m*−/*p*+^ neurons (*n* = 15 neurons/treatment group; Figure 7). A two-way ANOVA revealed a main effect of genotype (*F*_(1,84)_ = 7.87, *p* = 0.006), treatment (*F*_(2,84)_ = 3.22, *p* = 0.043), and an interaction (*F*_(1,2)_ = 4.96, *p* = 0.008). We did not observe a significant difference between baseline and BIC-induced Arc levels in WT neurons, although there was a trend towards higher levels. This may be due to already high basal neuronal activity levels. However, BIC treatment did significantly induce Arc expression over TTX-treated WT neurons (TTX: 69.8 ± 8.41, BIC: 112.88 ± 10.71; Tukey HSD, *p* = 0.023). However, in *Ube3a*^*m*−/*p*+^ neurons, BIC-induced Arc levels were not significantly different from TTX-induced (TTX: 70.74 ± 8.43, BIC: 56.26 ± 6.32; Tukey HSD, *p* = 0.9), and WT neurons expressed more Arc after BIC treatment than *Ube3a*^*m*−/*p*+^ neurons (Tukey HSD, *p* = 0.0007). These results show that Arc protein expression is uncoupled from neuronal activity in *Ube3a*^*m*−/*p*+^ neurons, which may lead to deficits in homeostatic scaling.

## Discussion

Here we show that, under multiple conditions, Arc and Ube3A do not physically interact *in vivo*. Despite a lack of interaction, we show for the first time that activity-dependent subcellular distribution of Arc is altered in *Ube3a*^*m*−/*p*+^ neurons, *in vivo*. Moreover, stability of Arc protein in dendrites is altered, which leads to deficits in homeostatic synaptic scaling of AMPARs, revealing an unknown synaptic deficit that may underlie the cognitive dysfunction in AS. These results suggest that Arc is not a direct E3 ligase substrate of Ube3A, and thus Ube3A controls Arc expression either through another direct substrate or a non-canonical E3 ligase function of Ube3A.

### Arc and *Ube3A* Do Not Interact *In Vivo*

Since the initial description of Arc as a substrate of Ube3A, two other studies (Kühnle et al., 2013; Mabb et al., 2014), as well as the present study (Figure 1), have shown that Ube3A and Arc do not directly interact, either by immunoprecipitation or *in vitro* binding methods. However, these two subsequent studies were performed under very disparate experimental paradigms: either using non-neuronal cells, or cultured cortical neurons that had been silenced for 24 h. Mabb et al. (2014) showed that Ube3A and Arc weakly coimmunoprecipitate from DIV21 cultured hippocampal neurons, although this finding was not replicated in HEK293 cells by the same group. Moreover, in the same study, the authors were unable to detect Ube3A-dependent ubiquitination of Arc. This was in contrast to another E3 ligase, Triad3A, which both robustly coimmunoprecipitated and ubiquitinated Arc (Mabb et al., 2014). Thus, we wanted to perform interaction studies in a more controlled and physiologically-relevant manner. Based on recent literature, we considered the possibility that the discrepancy may result from a transient interaction that occurs at different developmental ages and/or in different subcellular compartments. Arc is thought to act primarily at synapses, so we reasoned that immunoprecipitating from whole brain lysate might wash out a specific interaction found only in dendrites/synapses. However, we did not observe coimmunoprecipitation *in vivo* from subcellular compartments or at different ages. Moreover, we found that Ube3A is only weakly expressed at synapses under basal conditions. We did observe an increase in synaptically-localized Ube3A after animals experienced an enriched environment, but even under these conditions we were still unable to detect an interaction by coimmunoprecipitation. However, we cannot rule out that Arc and Ube3A transiently interact in dendrites under very spatially and temporally constrained conditions that our biochemical methods are unable to detect.

### Arc Protein Is More Stable in *Ube3a*^*m*−/*p*+^ Mice

Despite no evidence of an Arc-Ube3A interaction, our biochemical studies confirmed that Arc protein expression is dysregulated in *Ube3a*^*m*−/*p*+^ mice. Two previous studies found increases in Arc protein in *Ube3a*^*m*−/*p*+^ mice: in hippocampal lysates after stimulation (Greer et al., 2010), and in cultured cortical neurons after activity was suppressed for 24 h by TTX (Mabb et al., 2014). However, a different study found no difference in Arc protein from hippocampal lysates after stimulation or from BDNF-stimulated DIV7 cultured cortical neurons when comparing WT and *Ube3a*^*m*−/*p*+^ neurons (Mandel-Brehm et al., 2015). The conflicting findings are likely due to differences in mouse strain, *in vivo* vs. *in vitro*, age of culture, and experience or activity levels. We controlled for these variables in order to determine precisely how Arc expression is affected in *Ube3a*^*m*−/*p*+^ hippocampal neurons. Consistent with previous studies (Pignatelli et al., 2014; Mandel-Brehm et al., 2015), we found that the activity-dependent induction of Arc expression is normal in *Ube3a*^*m*−/*p*+^ neurons; thus, the most likely explanation for an increase in Arc protein is a lack of degradation leading to an increase in stability, and therefore an accumulation, of Arc protein. Interestingly, our results show that alterations in Arc protein stability are constrained to protein localized in dendrites/synapses, as little difference between genotypes was observed in the cytoplasm or nucleus (Figures 4, 5). Subcellular fractionation of hippocampal lysates from WT and *Ube3a*^*m*−/*p*+^ mice housed under basal conditions in the animal facility showed a similar ratio of Arc expression in the nucleus and synaptoneurosome compartments. However, after enriched environment, this ratio was dramatically different in the two genotypes. *Ube3a*^*m*−/*p*+^ mice had a much greater ratio of Arc in the synapse and in the nucleus. This might be due to overall higher levels of Arc in *Ube3a*^*m*−/*p*+^ mice, but also suggests that subcellular localization of Arc is aberrant in *Ube3a*^*m*−/*p*+^ mice. The results from these *in vivo* experiments corroborate the *in vitro* experiments, showing that Arc is specifically misregulated in dendrites and at synapses in *Ube3a*^*m*−/*p*+^ mice. Taken together, these results suggest that Ube3A may control dendritic levels of Arc in an activity-dependent manner. An alternative hypothesis is that Ube3A selectively regulates local translation of *Arc* in dendrites, rather than Arc protein stability, perhaps by regulating expression of another protein that controls Arc translation via upstream signaling pathways (Sell and Margolis, 2015). Known bona fide Ube3A substrates that may affect synaptic function include Ephexin5, which controls excitatory synapse formation (Margolis et al., 2010). A change in composition of excitatory synapses could lead to reduced *Homer1a* expression and enhanced mGluR5-LTD in *Ube3a*^*m*−/*p*+^ mice (Pignatelli et al., 2014). Local Arc translation is regulated by mGluR1/5 (Park et al., 2008; Waung et al., 2008), suggesting that misregulated or mislocalized Arc in *Ube3a*^*m*−/*p*+^ mice may contribute to this enhanced LTD phenotype.

### *Ube3a*^*m*−/*p*+^ Neurons Exhibit Disrupted Homeostatic Scaling

Arc is known to be critical for homeostatic synaptic scaling of AMPARs (Shepherd et al., 2006). Here, we found that homeostatic synaptic scaling of surface GluA1 receptors is disrupted in *Ube3a*^*m*−/*p*+^ neurons (Figures 6, 7), a novel cellular phenotype uncovered in our study. In a previous study, TTX-induced homeostatic scaling resulted in increased Arc expression in cultured cortical neurons from *Ube3a*^*m*−/*p*+^ mice as observed by Western blot (Mabb et al., 2014). Cultured hippocampal *Ube3a* full KO neurons exhibit reduced numbers of surface GluA1 puncta compared to WT cultures, under basal conditions (Greer et al., 2010), whereas we found surface GluA1 expression to be similar between WT and *Ube3a*^*m*−/*p*+^ neurons under basal conditions. The studies by Mabb et al. (2014) and Greer et al. (2010) underline the discrepancies and controversies relating to Arc expression: use of different strains of mice (full KO vs. deletion of the maternal allele only), focus on different brain regions (cortex vs. hippocampus), and experiments performed under varying levels of activity. In the present study, incubation with TTX or BIC for 24 h resulted in homeostatic scaling in WT neurons as shown by up- or downscaling of surface GluA1, but had no impact on surface GluA1 levels in *Ube3a*^*m*−/*p*+^ neurons. This is a similar phenotype to that seen in Arc KO neurons (Shepherd et al., 2006), highlighting the need for precise expression of Arc in the regulation of synaptic function. For example, *Ube3a*^*m*−/*p*+^ mice show an increase in spine elimination in the visual cortex during the critical period (Kim et al., 2016), a phenotype that would be expected from high levels of Arc (Peebles et al., 2010). While 24 h of TTX treatment decreased Arc expression in both WT and *Ube3a*^*m*−/*p*+^ neurons, at 10 h Arc expression was still much higher in *Ube3a*^*m*−/*p*+^ neurons (Figure 4). Thus, one explanation for a defect in TTX-induced upscaling in *Ube3a*^*m*−/*p*+^ neurons is slower clearance of Arc protein from synapses. Alternatively, Ube3A may play a role in homeostatic scaling in an Arc-independent manner, perhaps through unknown E3 ligase substrates. Twenty-four hours of BIC treatment increased Arc expression in WT neurons, but not *Ube3a*^*m*−/*p*+^ neurons, a possible mechanism for why downscaling is absent. Since induction of Arc is normal, we propose that the increase in Arc stability in *Ube3a*^*m*−/*p*+^ dendrites/synapses occludes BIC-dependent homeostatic scaling. Poor neuronal homeostasis may cause altered synaptic plasticity in *Ube3a*^*m*−/*p*+^ mice, since controlled expression of GluA1 at the surface of synapses is critical for proper LTP and depression (Shepherd and Huganir, 2007). Indeed, disruption of both LTP and LTD has been observed in *Ube3a*^*m*−/*p*+^ mice (van Woerden et al., 2007; Yashiro et al., 2009; Sato and Stryker, 2010; Pignatelli et al., 2014).

### Linking *Ube3A* and Arc Regulation

If Ube3A does not associate with Arc directly, how is Arc regulation altered in *Ube3a*^*m*−/*p*+^ neurons? *Ube3a*^*m*−/*p*+^ mice exhibit reduced CaMKII activity (Weeber et al., 2003). When mice genetically modified to have enhanced CaMKII activity were crossed with *Ube3a*^*m*−/*p*+^ mice, seizures, motor coordination and synaptic plasticity deficits were rescued (van Woerden et al., 2007). Arc and CaMKII are known to interact, and this interaction is thought to determine targeting of Arc to inactive synapses (Okuno et al., 2012). This suggests that the aberrant Arc regulation and localization in dendrites/synapses observed in the present study may be due to reduced CaMKII activity in *Ube3a*^*m*−/*p*+^ mice and not through direct Ube3A-dependent ubiquitination of Arc protein. However, it remains unclear whether CaMKII can directly phosphorylate Arc or whether an Arc-CaMKII interaction stabilizes Arc protein at synapses. Future studies will address the precise mechanistic link between Ube3A and the control of Arc protein expression.

## Conclusion

It is becoming apparent that neurodevelopmental disorders are caused by dysregulated synthesis of synaptic proteins. Many of these proteins are locally translated at synapses and are exquisitely regulated by multiple signaling pathways, including the mammalian target of rapamycin (mTOR) signaling (Huber et al., 2015). Poor neuronal homeostasis is a major basis of neurological diseases (Zoghbi and Bear, 2012). Arc lies at a critical nexus as a synaptic effector protein; disruption of Arc expression may therefore be detrimental for cognition. Since Arc expression is so tightly regulated, too little or too much Arc is harmful for normal synaptic function and cognition. Here we elucidate the synaptic plasticity deficits that may underlie the cognitive dysfunction observed in AS. Taken together, these studies suggest that deficits in Arc-dependent synaptic plasticity may be a common molecular pathway in genetic forms of intellectual disability that result from different genetic perturbations.

## Author Contributions

EDP performed experiments and analyzed and interpreted the data; JDS and EDP designed experiments, interpreted the data, and wrote the manuscript.

## Conflict of Interest Statement

The authors declare that the research was conducted in the absence of any commercial or financial relationships that could be construed as a potential conflict of interest.
